# Adherence to the Mediterranean Diet among School Children and Adolescents Living in Northern Italy and Unhealthy Food Behaviors Associated to Overweight

**DOI:** 10.3390/nu10091322

**Published:** 2018-09-18

**Authors:** Francesca Archero, Roberta Ricotti, Arianna Solito, Deborah Carrera, Federica Civello, Rosina Di Bella, Simonetta Bellone, Flavia Prodam

**Affiliations:** 1 SCDU of Pediatrics, Department of Health Sciences, University of Piemonte Orientale, 28100 Novara, Italy; francesca.archero@gmail.com (F.A.); robertaricotti@gmail.com (R.R.); arianna.solito@hotmail.it (A.S.); federicacivello@hotmail.it (F.C.); rosina.dibella@med.uniupo.it (R.D.B.); simonetta.bellone@med.uniupo.it (S.B.); 2 SCDO of Clinical Nutrition and Dietetics, Maggiore della Carità Hospital, 28100 Novara, Italy; carrera.deborah@gmail.com; 3 Interdisciplinary Research Center of Autoimmune Diseases, University of Piemonte Orientale, 28100 Novara, Italy; 4 Endocrinology, Department of Translational Medicine, University of Piemonte Orientale, 28100 Novara, Italy

**Keywords:** Mediterranean diet, questionnaire, children, adolescents, obesity

## Abstract

The purposes of this study were to evaluate the differences in Mediterranean diet and its components among primary and secondary school children and adolescents living in northern Italy, and the associations with the weight status. Adherence was assessed by the KIDMED (Mediterranean Diet Quality Index) questionnaire on 669 subjects (6–16 years) attending five schools of Novara. The adherence was poor in 16.7%, average in 63.7%, and high in 19.6% of the students. Poor adherence was more frequent in primary than in secondary schools (20.7% vs. 13.7%, *p* < 0.04). Some unhealthy behaviors were more prevalent in younger children. Children of other ethnic origins had a mixed behavior, choosing both traditional healthy and unhealthy foods. Besides male gender and primary school, in Italian children, the risk of overweight was directly associated with eating at fast-food restaurants (OR: 1.890, CI 95% 1.002–3.563), and inversely with consumption of vegetables more than once a day (OR: 0.588, CI 95% 0.349–0.991), and olive oil at home (OR: 0.382, CI 95% 0.176–0.826). In children of other ethnic origins, this risk was associated with skipping breakfast (OR: 16.046, CI 95% 1.933–133.266), or consuming commercial baked good or pastries for breakfast (OR: 10.255, CI 95% 1.052–99.927). The overall KIDMED score correlated with height (β: 0.108; *p* < 0.005). Poor food quality is replacing the Mediterranean dietary pattern in children and adolescents, in particular among younger children. Because the risk of overweight was associated with different components of the Mediterranean diet depending on ethnic origins, tailored nutritional programs remain a need.

## 1. Introduction

The Mediterranean diet (MD) is considered a model of a healthy diet, in particular after the publication of the first results derived by the PREDIMED study that demonstrated a reduction of cardiovascular mortality in subjects adherent to this dietary pattern [1]. MD has been associated with lower prevalence and/or incidence of several diseases, among others, type 2 diabetes, hypertension, cardiovascular diseases, and certain cancers that are all associated to overweight [2,3,4,5]. This is likely due to its composition rich in vegetables, fruits, legumes, whole cereals, as well as many sources of fiber and antioxidants, including fish, nuts, and extra-virgin olive oil. Moreover, the intake of sweets and trans fatty acids is low.

Although MD is beneficial, with the urbanization of people living in the Mediterranean area, in particular children and adolescents are deviating to a “Western diet” more rich in saturated fat, refined grains, simple carbohydrates and processed foods [6]. This phenomenon has been named nutrition transition and is one of the players implicated in the high prevalence of overweight and obesity in countries supposed to adopt a traditional MD [5,7]. A large meta-analysis of randomized controlled trials on MD reported a little but significant decrease in weight (−1.75 kg) and BMI (−0.57 kg/m^2^) in those adherents to MD [8]. Although intriguing results have been obtained in trials, the dissociation between higher obesity prevalence in Mediterranean countries and lower prevalence of many of its comorbidities in subjects adherent to a MD pattern is still an issue, in particular in pediatrics.

Studies investigating nutrition habits are needed to plan tailored strategies of interventions to educate on a healthy diet. In 2004, Serra-Majem et al. developed the KIDMED (Mediterranean Diet Quality Index for children and adolescents) score, a nutritional index validated in several languages that evaluates the adherence to MD and the quality of diet in children and adolescents [9]. Although it is simple to use in the clinical practice as well as in other epidemiological settings, only a few studies have been explored the adherence to the MD and to the risk of obesity in the young school population in Italy [10,11,12,13]. Most have been conducted in the southern part of Italy [14,15], where the obesity rate is higher than in other areas of the country [16], thus the generalizability has to be further demonstrated. The most extensive study using a modified version of the KIDMED score portrayed a school population of 1740 8–9-year-old children living in north Italy (Friuli, Venezia, Giulia and Liguria) and was conducted in 2009. The authors showed that only the 5.0% of the cohort was classified as high adherent to the MD, with the best rate (6.0%) in the north [17].

Although the benefits of the MD pattern can be considered as a synergistic interaction among all its components, a few studies reported protective or detrimental effects related to specific foods in adults with differences in prospective cohort or randomized controlled studies [5]. Vegetable intake was negatively associated, whereas higher intake of sweets, sugar-sweetened beverages, and fast foods was associated with obesity in a study on MD adherence conducted among adolescents living in Sicily [14]. No other Italian data have been published.

Based on the above, data on MD adherence are insufficient in children living in northern Italy. The first purpose of this study was to evaluate the differences in Mediterranean diet and its components among primary and secondary school children and adolescents living in Novara, a city of northern Italy characterized by an urban community employed in both agriculture and industry. The second purpose was to evaluated the associations of MD adherence and its components with the weight status.

## 2. Subjects and Methods

### 2.1. Population and Anthropometric Examination

This was a cross-sectional study conducted in April and May 2017. The study is a part of a cross-sectional study on pediatric obesity approved by the Ethical Committee of the Maggiore della Carità Hospital (CE 95/12).

We included 3 primary and 2 secondary schools of Novara. In 2017, the population of Novara was estimated at 104,183, with 18,634 people ≤14 years old. The average annual income per capita for the population is estimated around 16,132 Euro [18]. Before starting the enrollment, schools of Novara were classified according to socio-economic status based on estimates of the district’s socio-economic status in which they were located. We contacted all the schools by phone; to be selected they needed not to have developed a specific structured education program on MD in the year of recruitment. Eight schools respected all the inclusion criteria and were balanced for socio-economic status. Three of them refused to participate in the survey because no scholastic days were available. For all enrolled schools, all students attending all years were invited to participate with a letter carefully explaining the purpose of the study both to them and to their parents, and written informed consent was obtained. In addition, the children and adolescents provided their verbal assent on the day of the questionnaire. To be included in the analysis, participants should write and read fluently Italian.

In each school, data collection was performed by two pediatric nurses, one nutritionist, two physicians and a member of the department of the school policies who was responsible for the program. Questionnaires were completed during school hours in the classroom in the presence of a teacher, the nutritionist and at least one nurse and one physician. The staff helped with the questionnaire interpretation if needed. Questionnaires were anonymous. Students were requested to report their sex and date of birth. Nurses and physicians also performed the auxological examination after the completion of the questionnaire which returned at that moment. Anthropometric data were reported on the questionnaire form. Some days after the testing session a closing visit with a lesson on the MD and the MD food pyramid was conducted by the study staff.

Anthropometric measurements were performed in duplicate for each subject, wearing light indoor clothing and without shoes. Weight was measured to the nearest 100 g with a spring scale tested daily for accuracy and calibrated against a set of standard weights (Salus, Inc., Gaggiano, Milano, Italy). Height was measured with a standard laboratory stadiometer to the nearest 0.5 cm during maximal expiration. BMI was calculated as the ratio between weight (kg) and squared height (m^2^). BMI-SDS was calculated according to the LMS methods on the Italian charts [19]. Subjects were also stratified according to BMI categories (underweight, normal weight, overweight, obesity and morbid obesity) of the International Obesity Task Force [20].

Ethnicity was defined as the country of origin of the mother, in case of the different origin of both parents.

### 2.2. Evaluation of Adherence to the MD

We used the Italian version KIDMED index [21], a questionnaire of 16 dichotomous (positive/negative) items appropriate for youngsters. The answers with a positive connotation in relation to the MD are assigned a value of +1 (12 items), and those with a negative connotation, a value of −1 (4 items). The items explore the consumption of fruits, vegetables, fish, pasta/rice, cereals, yoghurt/cheese/dairy products, nuts, commercial baked and processed foods, breakfast habits and the frequency of skipping breakfast, fast-food frequency, sweet consumption, and olive oil during meals at home.

The overall score can range from −4 to 12. Total KIDMED scores were classified as follows: ≤3 reflects a poor adherence (very low diet quality), 4–7 an average adherence (improvement needed to adjust intake to MD patterns), and ≥8 a good adherence to the MD (optimal diet quality).

### 2.3. Statistical Analysis

Continuous data are expressed as mean, standard deviation (SD) and CI 95%. Prevalence of KIDMED, weight categories, and “yes” answers at each questionnaire item are reported as a percentage. The sample size was calculated according to the mean prevalence of low MD adherence according to the literature [8] with 95% confidence interval and an accuracy of ±4.0% of the average value of the adherence. A sample of 585 individuals was estimated as sufficient. Because the prevalence of obesity was relatively low, overweight and obese categories were considered together in the final analysis. Data were also stratified between primary and secondary schools. Socio-demographic level was defined according to that of the district area where the school was located. Kolmogorov–Smirnov test was used to test normality of variables’ distribution. Student’s independent t-test and Mann–Whitney U-test were used for normally and not normally distributed continuous variables, respectively. Two-tailed chi-square or Fisher exact test was used to evaluate differences in categorical variables, as appropriate. Univariate and multivariate logistic regression was used to assess the association of weight status with the odds ratio (OR, 95% CI) of gender, school level, ethnicity, MD adherence, or KIDMED items, as well as of MD adherence with gender, school level, ethnicity, and weight status. Because of several ethnic origins, ethnicity was categorized for statistical analyses as Italian and non-Italian. KIDMED items inserted in the models were those significant in the univariate analysis. Goodness-of-fit was evaluated by using the Hosmer and Lemeshow test; all the models were accepted because the χ^2^ was not significant. Interactions among variables (gender, school level, and ethnicity) were also explored; when *p* was >0.05 data in multinomial logistic analysis were only presented together. The KIDMED score and anthropometric parameters were also tested as continuous variables through linear regression stepwise analyses and the results are represented as standardized β coefficients. The level of statistical significance for analysis was set at *p* < 0.05. Statistical analysis was performed with SPSS for Windows version 17.0 (SPSS Inc., Chicago, IL, USA).

## 3. Results

### 3.1. Characteristics of the Population

The study was carried out in 669 subjects (324 males and 345 females) aged 11.2 (2.2) (CI 95% 11.0–11.4) years. All families and children gave their consent to participate in the study. Everybody completed the questionnaire and accepted the clinical visit. The analysis excluded six out of 705 subjects (0.9%) because they did not read and write fluently in Italian. All questionnaires were complete. The primary and secondary school samples were composed by 290 (138 males, 152 females) and 379 (186 males, 193 females) subjects, respectively. Six hundred twelve subjects (91.5%) were Italian. The remaining 57 subjects were born to parents from other ethnic origins. The majority of the subjects had a normal weight (*n* = 558; 83.4%); only 94 (14.1%) and 17 (2.5%) of them were overweight or obese, respectively. The prevalence of overweight plus obesity was higher in primary than in secondary schools (23.4% vs. 11.3%, χ^2^: 17.384, *p* < 0.0001), in males than females (21.3% vs. 12.2%, χ^2^: 10.047, *p* < 0.0001), and in subjects of other ethnic origins than those Italian (28.1% vs. 15.5%, χ^2^: 5.932, *p* < 0.001).

Sex, type of school, and ethnicity in the three-predictor model of OWB were all significant (model χ^2^: 31.971, *p* < 0.001) without interactions, accounting for 7.9% of the total variance (Nagelkerke *R*^2^), and the correct prediction rate was about 83.4%. In particular, the risk to be OWB was associated with male gender (OR: 2.024, CI 95% 1.322–3.099, *p* < 0.0001), primary school (OR: 2.387, CI 95% 1.562–3.648, *p* < 0.0001), and other ethnic origins (OR: 1.947, CI 95% 1.031–3.676, *p* < 0.03) in the corrected model. Table 1 represents demographic characteristics.

### 3.2. KIDMED

The adherence to the MD (scores ≤ 3) was poor in 16.7%, average (scores 4–7) in 63.7%, and high (scores ≥ 8) in 19.6% of the students. The overall score ranged from −1 to 11. The peak score was 6 (17.0%).

The prevalence rate of the three categories of adherence was similar between males and females, normal-weight (NW) and overweight/obese (OWB) subjects. The prevalence rate of poor adherence to the MD was significantly higher in primary than in secondary schools (20.7% vs. 13.7%, *p* < 0.04), with an equal rate of high adherence (19% vs. 20.1%). The four-predictor model of MD adherence (χ^2^: 8.000, *p* < 0.04) showed that only primary school was associated to a high risk of low MD adherence (OR: 1.618, CI 95% 1.068–2.452, *p* < 0.01), accounting for 2.1% of the total variance (Nagelkerke *R*^2^), and the correct prediction rate was about 83.3%. Table 2 shows the distribution of the levels of adherence among subgroups.

The risk of OWB previously described was not modified by the introduction in the model of the MD adherence either as category or as continuous variable.

The overall KIDMED score did not correlate with weight, BMI, and BMI SDS. Diversely, it correlated with height (β: 0.108; B: 0.015, CI 95% 0.005–0.026 *p* < 0.005) also when corrected for gender, age or school level, and ethnicity.

### 3.3. KIDMED Items

We analyzed the prevalence of “yes” answers in several subcategories.

Subjects with the lowest adherence to the MD answered “yes” less frequently in the positive questions, and more frequently in the negative ones (*p* < 0.0001) than those with the highest adherence to the MD. A similar distribution of answers was only reported on the daily intake of candy and sweets (Table A1).

In primary schools, children ate fewer vegetables once a day (χ^2^: 5.413, *p* < 0.01), fewer pulses once a week (χ^2^: 4.459, *p* < 0.02), and skipped breakfast (χ^2^: 5.375, *p* < 0.01). They also consumed more frequently pasta or rice almost every day (χ^2^: 4.4672, *p* < 0.01), ate at fast-food restaurants (χ^2^: 6.585, *p* < 0.007), consumed commercial baked good or pastries for breakfast (χ^2^: 17.034, *p* < 0.0001) or took sweets and candy several times every day (χ^2^: 18.610, *p* < 0.0001) than in secondary schools.

OWB consumed less olive oil (χ^2^: 4.704, *p* < 0.02), and more frequently ate at fast-food restaurants (χ^2^: 11.748, *p* < 0.001), skipped breakfast (χ^2^: 3.556, *p* < 0.04) or consumed commercial baked good or pastries for breakfast (χ^2^: 6.717, *p* < 0.006) than NW. Interestingly, OWB consumed more frequently vegetables once a day (χ^2^: 4.762, *p* < 0.01) or more than once a day (χ^2^: 4.000, *p* < 0.03) than NW.

Males skipped breakfast less (χ^2^: 3.187, *p* < 0.04) and consumed fish more regularly several times per week (χ^2^: 8.172, *p* < 0.003), but more frequently also ate at fast-food restaurants (χ^2^: 4.230, *p* < 0.02), and consumed commercial baked good or pastries for breakfast (χ^2^: 2.984, *p* < 0.04) than females.

Children of other ethnic origins consumed more fish (χ^2^: 6.460, *p* < 0.008), cereals or grain for breakfast (χ^2^: 3.705, *p* < 0.03), two yoghurts and/or some cheese (χ^2^: 6.083, *p* < 0.01) but more frequently also ate at fast-food restaurants (χ^2^: 5.505, *p* < 0.02), skipped breakfast (χ^2^: 6.621, *p* < 0.01), consumed commercial baked good or pastries for breakfast (χ^2^: 4.238, *p* < 0.05), or took sweets and candy several times every day (χ^2^: 6.847, *p* < 0.008) than those Italian.

Table 3 shows the distribution of subjects with respect to each item among subgroups.

In the crude analysis, the risk of OWB was associated with eating at fast-food restaurants (OR: 1.845, CI 95% 1.056–3.223, *p* < 0.03), frequent daily consumption of sweet and candies (OR: 1.946, CI 95% 1.238–3.059, *p* < 0.004), and in primary schools also with olive oil consumption at home (OR: 0.362, CI 95% 0.133–0.984, *p* < 0.04).

In the model weighted for all the items founded significant in the descriptive analysis, the risk of OWB (χ^2^: 57.393, *p* < 0.0001) was associated with the male gender (OR: 2.008, CI 95% 1.285–3.163, *p* < 0.002), the primary school (OR: 2.412, CI 95% 1.533–3.795, *p* < 0.0001), eating raw or cooked vegetables once a day (OR: 0.610, CI 95% 0.376–0.990, *p* < 0.04), olive oil consumption at home (OR: 0.521, CI 95% 0.255–0.973, *p* < 0.05), and consumed commercial baked good or pastries for breakfast (OR: 1.534, CI 95% 1.001–2.426, *p* < 0.05). The model accounted for 13.9% of the total variance (Nagelkerke *R*^2^), and the correct prediction rate was about 83.3%. We also split the analysis for the ethnicity due to the relative number of foreign children and significant interaction with some items. In Italian children, besides male gender and primary school, the risk of OWB (χ^2^: 54.208, *p* < 0.0001) was associated inversely with eating raw or cooked vegetables more than once a day (OR: 0.588, CI 95% 0.349–0.991, *p* < 0.04), and olive oil consumption at home (OR: 0.382, CI 95% 0.176–0.826, *p* < 0.01), and directly with eating at fast-food restaurants (OR: 1.890, CI 95% 1.002–3.563, *p* < 0.04). The model accounted for 14.2% of the total variance, and the correct prediction rate was about 84.2%. Diversely, in children of other ethnic origins, besides primary school, the risk of OWB (χ^2^: 22.201, *p* < 0.03) was associated with skipping breakfast (OR: 16.046, CI 95% 1.933–133.266, *p* < 0.01), or consuming commercial baked goods or pastries for breakfast (OR: 10.255, CI 95% 1.052–99.927, *p* < 0.03). The model accounted for 46.4% of the total variance, and the correct prediction rate was about 80.7%.

## 4. Discussion

Data on adherence to MD have been explored above all on Greek and Spanish pediatric populations. Italian data are relatively few and mainly referred to children aged 8–9 years, or adolescents living in the southern part of the country. We demonstrated that schoolchildren and adolescents, in particular primary school or overweight/obese students, are more likely to have dietary behaviors close to a Western dietary pattern. Moreover, pediatric subjects of other ethnic origins have mixed behaviors, as happens in the nutrition transition. Some specific unhealthy food choices are more prevalent, such as eating at fast-food restaurants, skipping breakfasts, consumption of commercially baked goods or pastries for breakfast, and of sweets several times every day. The risk of overweight/obesity was not associated with the overall adherence to MD, but with specific food habits different depending on ethnicity.

Firstly, we observed that the prevalence of overweight and obesity (17.6%) in our cohort was quite lower than that reported by the GBD 2015 Obesity Collaborators on children and adolescents younger than 20 years [22], the IDEFICS study on children aged 2–10 years [23], and the WHO European Childhood Obesity Surveillance Initiative on primary schoolchildren [24]. This result is likely due to the different age range in our school cohort (6–15 years), beyond a geographical reason being well known that the highest rate of overweight and obesity is in the southern part of Italy. Accordingly, the last Piedmont data derived by the “OKkio alla SALUTE” project on children aged 8–9 years are comparable with the prevalence (24.0% vs. 23.4%) observed in our primary schoolchildren and with a higher risk to be obese in males [16,25]. Moreover, we recorded a higher prevalence of overweight and obesity in children and adolescents of other ethnic origins than in Italian students. This finding is in agreement with data demonstrating that the prevalence of overweight and obesity has a negative gradient with social position and income across Europe [23].

Secondly, the overall prevalence of good adherence to the MD is less than 20%. Good adherence varies in the literature from 4.3% in Greek 10–12-year-old adolescents to 53.9% in Spanish children. Most of the studies conducted in southern European countries and recently reviewed reported that about half of pediatric individuals have an average adherence, while nearly half may have poor adherence [10,11]. Our data reflect those derived by the majority of the European studies [11]. Regarding Italy, our results are similar from the Calabrian Sierras Community Study (CSCS) which investigated a population attending primary and secondary schools in a 14-town southern Italian community [26]. We also observed a lower prevalence of good adherence in students attending primary than secondary school, suggesting that younger children are more subjected to unhealthy choices. In fact, the attendance at a primary school is the only significant risk factor related to a poor MD adherence. This result is in contrast with the majority of the data that reported a negative trend in MD adherence with age [10,11]. Unfortunately, the CSCS study did not stratify the data for the school level [27]. On the other hand, the prevalence in our secondary school sample is similar to that reported by other studies on adolescents living in southern Italy [14,25,27]. It has to been considered that other socio-demographic factors such as parents’ education and income are inconsistently associated across European countries due to different demographic and education changes [11]. Although we included only those schools where no prevention programs on the diets were performed in the last year, the enlargement of the study by including all the schools of Novara, accurate data on the socio-economic level, and parental weight could explain if an unexpected selection bias occurred.

Gender and overweight were not associated with the adherence to the MD. Although some studies suggest that in Western societies women tend to have better dietary habits than men [28], and MD has been associated to the prevention of obesity in adults [29], our results are in line with available European data in children recently systematically reviewed [10,11,30]. These findings suggest again that both obesity and social differentiation are complex events. Overeating, lack of physical activity, low sleep quality, and the family environment should be considered. On the other hand, the differences by gender, weight, and ethnicity on the items of the KIDMED index we recorded could help in explaining the phenomenon.

Our study reported a generally better quality of the diet among those children and adolescents more adherent to the MD than those with low adherence, except for the daily intake of sweets that was somewhat common. Moreover, we observed that younger children presented more unhealthy food choices than adolescents. This result is in line with a higher rate of poor adherence to the MD in the primary schoolchildren, as discussed above. The lower intake of vegetables and pulses resembles results obtained by the ZOOM8 study in 2009 and the last report of the “OKkio alla SALUTE” study in 2016 [16,31]. A negative association between MD adherence and snacks, sweets, commercial goods, and fast-foods has frequently been reported in other European pediatric populations [10,11,16]. It is interesting that primary schoolchildren consumed a more Westernized diet, skipping breakfast, eating several times per week at fast-food restaurants, consuming commercial baked goods, and sweets. This phenomenon could be boosted by geographical reasons, being our study conducted in an urban area [25]. In line with this hypothesis, we recorded a mixed dietary behavior in children and adolescents of other ethnic origins. In fact, the latter maintained a higher intake of more traditional foods, such as fish, cereals or grain for breakfast, and yoghurts and/or some cheese, suggesting more home-made foods in their family environment. On the other hand, contemporarily, they frequently ate at fast-food restaurants, skipped breakfast, consumed commercial baked goods for breakfast, or sweets and candy several times every day. These findings well picture the nutrition transition described in European countries including those of the Mediterranean area [7,11]. Overeating, eating anything or disliked foods, and eating at friends’ home were all identified as strategies to cope with food insecurity [32]. Frequent consumption of fast food/junk calorie dense foods have been reported in several developing countries [33], and these behaviors could be replicated when low-income families move abroad, and less control over the youngest generations occurs for several reasons [11].

As previously described, the adherence to the MD did not predict the weight status. Moreover, in the literature, there is inconsistency also in the evidence about the role of specific food groups [34]. However, we recorded some associations with single KIDMED items. First, the risk of being OWB was related to eating at fast-food restaurants and daily intake of commercial baked goods or pastries for breakfast. This result is in agreement with two systematic reviews, one reporting the role of ultra-processed foods and the other that of sugar-sweetened drinks and sweets snacking with increased obesity risk also in the pediatric populations [35,36]. Direct associations between fast food availability and obesity in lower-income children have been described [37]. The adherence to MD depends on many foods with specific and synergistic activities on metabolism. It is likely that dense energy foods rich in simple carbohydrates and saturated fats have a major role in the development of obesity, in particular in younger children.

Moreover, food habits associated with the risk of OWB are different depending on ethnic backgrounds. The higher risk of OWB in those of other ethnic origins related to unhealthy choices for breakfast with skipping it or consuming commercial sweets and pastries suggests that the urbanization of life may lead to a more stressful lifestyle also in migrant people with less time spent on cooking, more time out of home, and dinner as the principal meal consumed with the family. All these factors could influence the food choices of the youngest children.

Interestingly, OWB subjects consumed less olive oil at home, and the risk of OWB was significantly and inversely associated with the intake of olive oil. The fact that the olive oil intake is associated with BMI is still debated. A study in children observed that the likelihood to increase their BMI was less in those who consumed only olive oil than in those who consumed other oils [38]. A recent review did not observe an increase in weight with an enriched-olive oil diet [39], although the Food4Me study recently recorded a direct correlation with the increase in weight in adults [34]. However, these last data are a little bit ambiguous reporting at the same time an inverse relationship with the intake of monounsaturated fats.

Furthermore, OWB children and adolescents consumed more frequently vegetables than NW ones. This finding could be contrasting with their other food habits. However, the risk of OWB was inversely associated with the intake of vegetables more than once a day. In addition, in the ZOOM study, OWB subjects consumed more vegetables than NW children [30], suggesting that the answers to these items hide an overeating behavior that overcomes the protective effects of healthy dietary choices [40].

Finally, we observed a direct association between the KIDMED score and height. To our knowledge, this result has not been reported by other studies on the adherence to the MD, because they have been focused on BMI, waist or waist-to-height without considering height alone [10,11], with the exception of one study limited to nine-year-old children [41]. Since children with a medium/high adherence to the MD have a more balanced diet in terms of nutrients and functional foods [10], these habits can explain our data. In particular, a diet poor in micronutrients and high-quality proteins from milk products, pork meat, and fish has been shown to negatively influence stature [42,43,44]. The reanalysis of data derived by HELENA and IDEFICS cohorts could confirm our findings.

This study has some limitations. First, it was a cross-sectional study design. Therefore, it is a limited set to establish causal relationships between MD and health outcome, and then conclusions are indications for further prospective and experimental investigations. Second, we only used the KIDMED score, without integrating it with a food frequency questionnaire. Adherence indexes, such as KIDMED score, have been validated and used in epidemiological surveys, but their reliability and reproducibility in assessing diet quality in the single subject have not been demonstrated yet [10,11]. Indeed, data on diet composition related to KIDMED score can be only inferred from related limited literature. We cannot exclude that some nutrients are main players more than food habits we reported. On the other hand, we used the most used index of adherence in pediatric literature [10,11], and the precise and driven administration to our cohort, in particular to younger children, supports the accuracy of our data. In fact, some authors suggest that the variability of adherence to MD across studies also results from the different administration methodologies [10]. Third, data on physical activity and sleep quality are lacking. Sedentary behaviors have been demonstrated to be correlated with the risk of OWB and low adherence to MD [10,11], and, then, co-linearity with some variable could exist. Fourth, specific data on the socio-economic level were not obtained, and schools were only stratified according to the socio-economic level of the district area they were located, and an unexpected selection bias could have occurred. Indeed, more accurate investigations of these variables, including also parental education, could have a role and improve the prediction of the OWB risk. On the other hand, this study could give a significant contribution to research since recent data on northern Italy are lacking and it could be compared with similar studies conducted in the Southern part of Italy, as well as in other urban European areas. We presented data divided for school level, gender and ethnicity and this will help in making more effective reviews on the topic. In particular, we focused on how different food habits influence the risk of OWB depending on ethnicity. These data are crucial for further investigations and description of the nutrition transition phenomenon with urbanization. Moreover, weight and height were not self-reported, and this increased the accuracy of the relationship between weight status and MD adherence.

In general, our study confirms that both children and adolescents have a poor MD adherence in an urban area of northern Italy, in line with other Italian and European data [10,11]. Furthermore, the mixed food behaviors occurring in individuals of different ethnic origins suggests that tailored prevention programs are needed to mitigate in this category of people the nutrition transition resulting from urbanization and changes of lifestyle habits of their families. These programs are urgent in hopes of preserving traditional healthy food habits. Moreover, the risk of OWB seems directly and indirectly associated more with specific food categories in pediatrics. These results should be confirmed but suggest that we have to increase nutrition knowledge in children and parents, as well as nutrition researchers should work hard.

## 5. Conclusions

In conclusion, we observed a relatively low high adherence to the MD in children and adolescents, in particular in those attending primary schools. Skipping breakfast, eating at fast-food restaurants, intake of processed foods and sweets are the main unhealthy choices, in particular in those OWB or of other ethnic origins. Differences in adherence to the MD and food intake between primary and secondary school, NW and OWB subjects and ethnic groups should be taken into account. Strategies tailored explicitly to subgroups are needed. Prevention campaigns should be conducted to improve food quality and drive to consume home-made healthy foods. The phenomenon of the nutrition transition should be accurately investigated, in particular in younger children and those in low-income brackets.

## Figures and Tables

**Table 1:** Anthropometric characteristics of the 669 subjects, by school level.

	All	Primary School	Secondary School	*p*
Gender				0.755
M	324 (48.4%)	138 (47.5%)	186 (49.0%)	
F	345 (51.6%)	152 (52.5%)	193 (51.0%)	
Age (years)	11.2 (2.2)	9.0 (1.3)	12.9 (1.1)	0.0001
	(11.0–11.4)	(8.8–9.2)	(12.8–13.1)	
Ethnicity				0.03
Italian	612 (91.5%)	258 (89.0%)	354 (93.4%)	
Eastern European	22 (3.3%)	7 (2.4%)	15 (4.0%)	
African	25 (3.7%)	22 (7.6%)	3 (0.8%)	
Asian	4 (0.6%)	1 (0.3%)	3 (0.8%)	
South American	6 (0.9%)	2 (0.7%)	4 (1.0%)	
Height (cm)	147.9 (15.6)	134.6 (10.3)	158.2 (10.5)	0.0001
	(146.8–149.2)	(133.4–135.8)	(157.1–159.3)	
Weight (Kg)	40.4 (12.6)	31.3 (7.6)	47.4 (11.2)	0.0001
	(39.5 41.4)	(30.5 32.3)	(46.3–48.5)	
BMISDS	−0.476 (1.028)	−0.309 (1.076)	−0.604 (0.972)	0.0001
	(−0.554, −0.398)	(−0.433, −0.185)	(−0.703, −0.507)	
BMI category				0.0001
Normal-weight	558 (83.4%)	222 (76.5%)	336 (88.6%)	
Overweight	94 (14.1%)	57 (19.7%)	37 (9.8%)	
Obese	17 (2.5%)	11 (3.8%)	6 (1.6%)	

Data are expressed as mean ± SD, CI 95%, absolute numbers and percentages. Differences among categorical variables were tested by Chi-square test. Associations between variables were tested by Student’s independent t-test (BMISDS), or Mann–Whitney U-test (age, height, and weight). Abbreviations: F, female; M, male. BMI was stratified according to the IOTF criteria.

**Table 2:** Distribution of the adherence to the MD of the study population and relative odd ratios among subcategories.

	KIDMED Score	*p*	KIDMED Score Low	KIDMED Score Medium	KIDMED Score High	*p*	Adj OR (CI 95%) of High Adherence
School	5.4 (2.3)	0.094				0.04	
Primary	(5.1 5.7)		60 (20.7%)	175 (60.3%)	55 (19.0%)		1
Secondary	5.6 (2.1)		52 (13.7%)	251 (66.2%)	76 (20.1%)		1.618
	(5.5–5.9)						(1.068–2.452)
Weight	5.6 (2.1)	0.364				0.610	
NW	(5.4–5.8)		91 (16.3%)	359 (64.3%)	108 (19.4%)		1
OWB	5.6 (2.4)		21 (18.9%)	67 (60.4%)	23 (20.7%)		0.868
	(4.9–5.9)						(0.504–1.494)
Gender	5.6 (2.1)	0.062				0.062	
M	(5.5–5.9)		47 (14.5%)	209 (64.5%)	68 (21.0%)		1
F	5.4 (2.2)		65 (18.8%)	217 (62.9%)	63 (18.3%)		0.725
	(5.2–5.7)						(0.478–1.098)
Ethnicity	5.5 (2.1)	0.092				0.672	
Italian	(5.4–5.8)		103 (16.8%)	389 (63.6%)	120 (19.6%)		1
Others	5.5 (2.2)		9 (15.8%)	37 (64.9%)	11 (19.3%)		0.850
	(5.0–6.1)						(0.400–1.494)
All	5.5 (2.1)	//	112 (16.7%)	426 (63.7%)	131 (19.6%)	//	//
	(5.4–5.7)						

KIDMED score as continuous variables is expressed as mean (SD) and CI 95%. Adjusted Odd Ratios (OR) were calculated by binary logistic regression analysis with low adherence as reference category in dependent variable, and school level, gender, weight status, and ethnicity as independent variables. Medium and high adherences were considered together. The ORs were referred to secondary school, female gender, OWB, and other ethnic origin. Abbreviations: F, female; M, male; NW, normal-weight; OWB, overweight + obese. //: not calculable.

**Table 3:** Distribution of “yes” answers by school level, weight status, gender, and ethnicity.

	School Level		Weight		Gender		Ethnicity	
	Primary School	Secondary School	NW	OWB	M	F	Italian	Other
Consumption of a fruit or a fruit juice every day ^1^	215 (74.1%)	292 (77.0%)	426 (76.3%)	81 (73.0%)	253 (78.1%)	254 (73.6%)	462 (75.5%)	45 (78.9%)
Consumption of a second fruit every day ^1^	141 (48.6%)	180 (47.5%)	264 (47.3%)	57 (51.4%)	153 (47.2%)	168 (48.7%)	291 (47.5%)	30 (52.6%)
Consumption of raw or cooked vegetables 1 time a day ^1^	**146** **(50.3%)**	**225** **(59.4%)**	**299** **(50.3%)**	**72** **(64.9%)**	181 (55.9%)	190 (55.1%)	335 (54.7%)	36 (63.2%)
Consumption of raw or cooked vegetables >1 time a day ^1^	79 (27.2%)	121 (31.9%)	**158** **(28.3%)**	**42** **(37.8%)**	101 (31.2%)	99 (28.7%)	185 (30.2%)	15 (26.3%)
Consumption of fish regularly (at least 2–3 times a week) ^1^	138 (47.6%)	177 (46.7%)	256 (45.9%)	59 (53.2%)	**171** **(52.8%)**	**144** **(41.7%)**	**279** **(45.6%)**	**36** **(63.2%)**
Eating >1 time per week to a fast-food (hamburger) restaurant ^2^	**57** **(19.7%)**	**47** **(12.4%)**	**75** **(13.4%)**	**29** **(26.1%)**	**60** **(18.5%)**	**44** **(12.8%)**	**89** **(14.5%)**	**15** **(26.3%)**
Consumption of beans >1 time per week ^1^	**150** **(51.7%)**	**227** **(56.7%)**	313 (56.1%)	64 (57.7%)	189 (50.1%)	188 (49.9%)	347 (56.7%)	30 (52.6%)
Consumption of pasta or rice almost every day (≥5 times a week) ^1^	**251** **(86.6%)**	**304** **(80.2%)**	466 (83.5%)	89 (80.2%)	267 (82.4%)	288 (83.5%)	510 (83.3%)	45 (78.9%)
Consumption of cereals or grains (bread, etc.) for breakfast ^1^	166 (57.2%)	223 (58.8%)	328 (58.8%)	61 (55.0%)	197 (60.8%)	192 (55.7%)	**349** **(57.0%)**	**40** **(70.2%)**
Consumption of nuts regularly (at least 2–3 times per week) ^1^	75 (25.9%)	92 (24.3%)	134 (24.0%)	33 (29.7%)	80 (24.7%)	87 (25.2%)	149 (24.3%)	18 (31.6%)
Consumption of olive oil at home ^1^	264 (91.0%)	354 (93.4%)	**521** **(93.4%)**	**97** **(87.4%)**	300 (92.6%)	318 (92.2%)	567 (92.6%)	51 (89.5%)
Skipping breakfast ^2^	**43** **(14.8%)**	**83** **(21.9%)**	**98** **(17.6%)**	**28** **(25.2%)**	**52** **(16.0%)**	**74** **(21.4%)**	**108** **(17.6%)**	**18** **(31.6%)**
Consumption of a dairy product for breakfast (yoghurts, milk, etc.) ^1^	225 (77.6%)	275 (72.6%)	422 (75.6%)	78 (70.3%)	248 (76.5%)	252 (73.0%)	459 (75.0%)	41 (71.9%)
Consumption of commercially baked goods or pastries for breakfast ^2^	**182** **(62.8%)**	**117** **(46.7%)**	**287** **(51.4%)**	**72** **(64.9%)**	**185** **(57.1%)**	**174** **(50.4%)**	**321** **(52.5%)**	**38** **(66.7%)**
Consumption of 2 yoghurts and/or cheese (40 g) daily ^1^	114 (39.3%)	170 (44.9%)	236 (42.3%)	48 (43.2%)	144 (44.4%)	140 (40.6%)	**251** **(41.0%)**	**33** **(57.9%)**
Consumption of sweets or candy several times every day ^2^	**154** **(53.1%)**	**138** **(36.4%)**	**287** **(51.4%)**	**72** **(64.9%)**	190 (58.6%)	187 (54.2%)	**258** **(42.2%)**	**34** **(59.6%)**

Numbers and percentages are referred to “yes” answers. The denominators are those described in Table 1. ^1^ Items with a positive answer (+1). ^2^ Items with a negative score (−1). Bold numbers are those significant in the univariate logistic regression. Abbreviations. F, female; M, male; NW, normal-weight; OWB, overweight + obese.

## Appendix A

**Table A1:** Comparison of the each KIDMED item in children with the lowest (score ≤ 3) and the highest adherence (score ≥8) to the Mediterranean diet and worst lifestyle.

	KIDMED Score Low (*N* = 112)	KIDMED Score High (*N* = 131)	*p*
Consumption of a fruit or a fruit juice every day ^1^	45.5%	83.1%	0.0001
Consumption of a second fruit every day ^1^	17.0%	87.0%	0.0001
Consumption of raw or cooked vegetables 1 time a day ^1^	17.9%	87.0%	0.0001
Consumption of raw or cooked vegetables >1 time a day ^1^	9.8%	64.1%	0.0001
Consumption of fish regularly (at least 2–3 times a week) ^1^	16.1%	73.3%	0.0001
Eating >1 time per week to a fast-food (hamburger) restaurant ^2^	37.5%	9.2%	0.0001
Consumption of beans >1 time per week ^1^	24.1%	84.7%	0.0001
Consumption of pasta or rice almost every day (≥5 times a week) ^1^	66.1%	88.5%	0.0001
Consumption of cereals or grains (bread, etc.) for breakfast ^1^	23.2%	85.5%	0.0001
Consumption of nuts regularly (at least 2–3 times per week) ^1^	14.3%	48.9%	0.0001
Consumption of olive oil at home ^1^	77.7%	100.0%	0.0001
Skipping breakfast ^2^	37.5%	5.3%	0.0001
Consumption of a dairy product for breakfast (yoghurts, milk, etc.) ^1^	42.9%	95.4%	0.0001
Consumption of commercially baked goods or pastries for breakfast ^2^	66.1%	37.4%	0.0001
Consumption of 2 yoghurts and/or cheese (40 g) daily ^1^	32.1%	56.5%	0.0001
Consumption of sweets or candy several times every day ^2^	50.0%	40.5%	0.087

^1^ Items with a positive answer (+1). ^2^ Items with a negative score (−1). Data are represented as percentages. The data were analyzed by univariate logistic regression.
